# Prevalence of diabetes and insulin resistance in patients with diagnosis of schizophrenia or other psicotic disorders

**DOI:** 10.1192/j.eurpsy.2024.1540

**Published:** 2024-08-27

**Authors:** P. Andres-Olivera, J. Seabra de Brito, B. Arribas-Simon, C. Martin-Gomez, B. Bote, C. Payo, C. Munaiz, R. Brito, M. Ligero-Argudo, D. Jimenez Martinez, C. Roncero

**Affiliations:** ^1^Psychiatric Service, University of Salamanca Health Complex; ^2^Psychiatric Unit. School of Medicine, University of Salamanca; ^3^PRINT, Biomedical Research Institute (IBSAL), Salamanca; ^4^Psychiatric Service, University of Valladolid Healthcare Complex, Valladolid; ^5^Biomedical Research Institute (IBSAL), Salamanca, Spain

## Abstract

**Introduction:**

Contrary to classical belief, people affected by this disease are at greater risk of developing organic pathologies.This risk has a very complex origin: a greater exposure to risk factors and specific socioeconomic conditions, a high prevalence of risk behaviors, the use of antipsychotics, and a potential common genetic background. (Reynolds *et al*.Int. J.Neuropsychopharmacol.2021; 24 854–855, *Suvisaari J et al.* Curr Diab Rep. 2016 16). Multiple studies demonstrate that Schizophrenia confers a high endogenous risk of Diabetes. Before patients diagnosed with Schizophrenia start taking antipsychotics (Andreassen OA *et al.* Am J Psychiatry. 2017;174 616-617), they have an approximately 3 times higher risk of developing Diabetes compared to the general population. The risk increases 3.6 times after the initiation of antipsychotic treatment compared to drug naive patients(*Annamalai A et al* World J Diabetes. 2017 390-396)

**Objectives:**

To study the association between Schizophrenia or other Psychotic Disorders and Diabetes Mellitus in a sample of patients diagnosed with Schizophrenia or other Psychotic Disorders.

**Methods:**

This is a Descriptive and Cross-sectional Observational Study. Clinical Histories were reviewed and a personal or telephone interview was established to expand data related to the objectives of the study. The patients were recruited among the patients seen in the specific Severe Mental Disorder consultation who had a diagnosis of schizophrenia or other Psychotic Disorders, according to DSM 5-TR criteria.

**Results:**

From a sample of 93 patients, 24 had Diabetes. The Prevalence of Diabetes in patients with Schizophrenia or other Psychotic Disorders was 25.8%. Of the patients without a diagnosis of Diabetes, 15 of them had values of Glycosylated Hemoglobin (HbA1c) for Prediabetes. Using the Chi-Square Test, statistically significant differences were found between the variable Main Psychiatric Medication and Diabetes. Patients treated with Clozapine, Aripiprazole and Olanzapine had a Prevalence of Diabetes of 40.9%, 33.3% and 28.5%, respectively.

**Conclusions:**

Prevalence of Diabetes in our sample was 3.4 times higher than the 7.51% of the general population in Spain. This presumes a significant importance and impact on the health of these patients. The diabetic patients in our sample were diagnosed with Diabetes years after the diagnosis of the mental illness, which seems to indicate that the causes have to do with lifestyle, dietary habits, weight, and exposure to chronic antipsychotics. Premature death in schizophrenia has several explanations, being of special importance the development of cardiovascular disorders and Diabetes This can be due to many reasons, but it is worth highlighting the metabolic side effects of some antipsychotics and lifestyle. In this sense, it is essential to carefully monitor this group of patients.

**Disclosure of Interest:**

None Declared

